# Verona Integron–encoded Metallo-β-Lactamase 1 in Enterobacteria, Ontario, Canada

**DOI:** 10.3201/eid1907.121294

**Published:** 2013-07

**Authors:** Nathalie Tijet, Gregory Macmullin, Olga Lastovetska, Christie Vermeiren, Patricia Wenzel, Tina Stacey-Works, Donald E. Low, Samir N. Patel, Roberto G. Melano

**Affiliations:** Public Health Ontario Laboratories–Toronto, Toronto, Ontario, Canada (N. Tijet, G. Macmullin, O. Latovetska, D.E. Low, S.N. Patel, R.G. Melano);; University of Toronto, Toronto (O. Lastovetska, D.E. Low, S.N. Patel, R.G. Melano);; Shared Hospital Laboratory, Toronto (C. Vermeiren);; Halton Healthcare Services, Oakville, Ontario, Canada (P. Wenzel, T. Stacey-Works);; Mount Sinai Hospital, Toronto (D.E. Low, R.G. Melano)

**Keywords:** VIM-1, Verona integron-encoded metallo-β-lactamase 1, Enterobacteriaceae, enterobacteria, Escherichia coli, Enterobacter cloacae, bacteria, antimicrobial resistance, Ontario, Canada, Enterobacteria

**To the Editor:** Among *Enterobacteriaceae*, Verona integron–encoded metallo-β-lactamase 1 (VIM-1) has been found only in *Klebsiella pneumoniae* in North America (*1*). We report 4 VIM-1–producing *Enterobacteriaceae* isolated from 4 patients at 3 hospitals in Ontario, Canada.

Patient 1, a 61-year-old man, was initially hospitalized in Italy for presumed pneumonia and was treated with levofloxacin during his 6-month stay in Italy. Upon returning to Ontario, Canada, he was admitted to hospital 1 in August 2010 because of diabetic ketoacidosis and began empiric treatment with metronidazole and gentamicin. Urine cultures were positive for a carbapenem-resistant *Escherichia coli* (strain GN531). Two days later, the patient had a fever and a blood culture was positive for *E. coli* (strain GN532), which was also resistant to carbapenems. During his hospitalization, the patient was isolated and received droplet precaution because of his travel history until he was discharged home.

Patient 2, a 76-year-old man, was admitted to hospital 2 in May 2011 because of a recurrent urinary tract infection (urine was positive for *E. coli*). The patient was given ciprofloxacin. On day 49, a carbapenem-sensitive *Enterobacter cloacae* was isolated from urine. On day 61, a carbapenem-resistant *E. cloacae* was isolated from urine culture (strain GN719). Contact precautions were used until the patient was discharged to a long-term care facility on day 80.

Patient 3, an 81-year-old man, was admitted to hospital 2 (November 2011) 2 months after patient 2 was discharged. Urine culture at admission was positive for a carbapenem-resistant *E. cloacae* (strain GN825). The patient was given ceftriaxone and metronidazole and then given ertapenem. The patient died on day 110. Patients 2 and 3 had no hospital room in common during their admissions and both received contact precautions for methicillin-resistant *Staphylococcus aureus* before isolation of the carbapenem-resistant isolates.

Patient 4, a 90-year-old woman, was admitted to hospital 3 in November 2011 because of nausea, vomiting, and diarrhea. In the preceding 6-month period, she had recurrent *Clostridium difficile*–associated diarrhea and a urinary tract infection. At admission, a carbapenem-susceptible *Proteus* spp. was isolated from a urine culture. The patient was given a 3-day course of ciprofloxacin and vancomycin. On day 17, a carbapenem-resistant *E. cloacae* was isolated from urine (strain GN738). Because this organism was also isolated from a rectal swab specimen, it was assumed that the urine sample might be contaminated by her feces. Therefore, the patient did not receive additional treatment other than that for recurrent *C. difficile*–associated diarrhea.

Patients 2, 3, and 4 had no history of travel outside Canada. All 5 isolates were submitted for reference purposes to the Public Health Ontario Laboratories. Pulsed-field gel electrophoresis showed that *E. coli* GN531 and GN532 were indistinguishable (GN531 was selected for further studies), and the 3 *E. cloacae* isolates had similar fingerprint patterns. All strains displayed synergy in presence of meropenem disks plus dipicolinic acid, which is indicative of metallo-β-lactamase inhibition (*2*). The 4 clinical strains displayed a multidrug resistance phenotype, and were susceptible only to tigecycline and colistin (Table).

**Table:** VIM-1–producing *Escherichia coli* and *Enterobacter cloacae* clinical isolates, derivative transconjugants, and transformants, Ontario, Canada*

Characteristic	*E. coli* GN531	*E. cloacae* GN719	*E. cloacae* GN738	*E. cloacae* GN825	*E. coli* J-531	*E. coli* T-719	*E. coli* T-825	*E. coli *Top10	*E. coli* J53
Drug, MIC (mg/L)†									
Ampicillin	≥256	≥256	≥256	≥256	≥256	≥256	≥256	3	6
Cefoxitin	≥256	≥256	≥256	≥256	64	≥256	≥256	6	8
Ceftazidime	≥256	≥256	≥256	≥256	≥256	≥256	≥256	0.19	0.19
Cefotaxime	≥256	≥256	≥256	≥256	96	128	≥256	0.094	0.094
Cefepime	256	32	192	256	12	12	24	<0.016	0.064
Ertapenem	2	2	8	24	0.125	0.25	0.25	0.004	0.008
Meropenem	1.5	6	6	16	0.5	0.5	0.5	0.023	0.023
Imipenem	4	6	6	8	2	2	1.5	0.19	0.38
Aztreonam	≥256	0.19	4	1.5	192	0.125	0.125	0.125	0.125
Amikacin	8	2	3	2	3	1.5	1.5	2	1.5
Gentamicin	96	12	2	96	4	0.75	2	0.064	1.5
Tobramycin	32	48	6	32	12	4	4	0.25	1
Nalidixic acid	≥256	≥256	≥256	≥256	32	2	1	1	3
Ciprofloxacin	≥32	≥32	≥32	≥32	0.5	0.125	<0.002	<0.002	0.012
Levofloxacin	≥8	≥32	≥32	8	0.5	0.094	0.002	0.003	0.016
Tetracycline	≤4	192	2	256	0.5	32	32	0.75	1
Tigecycline	0.094	1	0.5	1	0.047	0.064	0.094	0.032	0.047
Colistin	0.064	0.094	0.094	0.125	0.047	0.023	0.016	0.016	0.047
Co-trimoxazole	≥32	≥32	≥32	≥32	≥32	≥32	0.047	0.023	0.064
Drug resistance gene‡									
* bla*_VIM-1_	*+*	*+*	*+*	*+*	*+*	*+*	*+*	NA	NA
* bla*_CTX-M-15_	*+*	–	–	–	–	–	–	NA	NA
* bla*_TEM-1_	–	*+*	–	+	–	–	–	NA	NA
* bla*_ACC-1_	–	–	*+*	*+*	–	–	–	NA	NA
* bla*_OXA-1 like_	*+*	–	–	–	–	–	–	NA	NA
* bla*_SHV-12_	*+*	–	–	–	+	–	–	NA	NA
* qnrS1*	*+*	+	+	–	+	+	–	NA	NA
Replicon type§									
IncN	+	+	+	+	+	+	–	NA	NA
IncFrep	+	+	–	+	–	–	–	NA	NA
IncFIB	+	–	–	+	–	–	–	NA	NA
IncFIA	+	–	–	–	–	–	–	NA	NA

*VIM-1, Verona integron–encoded metallo-β-lactamase 1; *E. coli* J-531, *E. coli* transconjugant derived from GN531; *E. coli* T-719 and T-825, *E. coli* transformants derived from GN719 and GN825, respectively; *E*. *coli* J53 and TOP10, recipient *E. coli* J53 and TOP10, respectively; bla, β-lactamase; +, positive; NA, not applicable (only genes and replicons detected by molecular screening are included); –, negative; qnr, quinolone resistance; Inc, incompatibility. †Drug susceptibility results were determined by using Etest (bioMérieux, Marcy l’Etoile, France) and the agar dilution method and interpreted by using Clinical and Laboratory Standards Institute guidelines (*3*). ‡Sequencing of whole genes was performed in samples positive by PCR. PCR included screening for *bla*_TEM_; *bla*_SHV_; *bla*_OXA-1-like_; *bla*_CTX-M_ groups 1, 2, and 9; *bla*_VEB_; *bla*_PER_; *bla*_GES_; *bla*_OXA-48-like_; *bla*_IMP_; *bla*_KPC_; *bla*_NDM-1_; and 6 groups of *bla*_AmpC_ genes (*4*). §Obtained by using the replicon typing approach of Carattoli et al. (*5*).

PCR and sequencing identified *bla*_VIM-1_ in all isolates (Table). Multilocus sequence typing classified *E. coli* GN531 as sequence type (ST) 131 (*6*), the epidemic strain that spreads *bla*_CTX-M-15_ worldwide (*7*). *E. coli* ST131 with similar phenotypic and genetic features was described in Florence, Italy, in 2009 (*8*). Because *E. coli* GN531 was isolated from patient 1, who had received heath care in Italy before being hospitalized in Ontario, this patient might have been exposed to this strain in Italy. A similar scenario was reported in the first case of VIM-1–producing *K. pneumoniae* in the United States, which was isolated from a patient who received health care in Greece (*1*). The presence of a metallo-β-lactamase in *E. coli* ST131 is of great concern because it increases the potential for dissemination of drug-resistance genes.

An IncN plasmid (*5*) harboring *bla*_VIM-1_ was transferred from GN531 to *E. coli* by conjugation (Table). The *bla*_CTX-M-15_ gene was not co-transferred, which indicated that it was located on another plasmid or the chromosome of the clinical isolate. After several attempts, no transconjugants derived from *E. cloacae* were obtained. *E. coli* TOP10 (Life Technologies, Carlsbad, CA, USA) was transformed with VIM-1 plasmids obtained from *E. cloacae* GN719 and GN825 (T-719 and T-825, respectively). *E. coli* transformation with plasmid extracts from *E. cloacae* GN738 was unsuccessful. Pulsed-field gel electrophoresis with S1 nuclease (*9*) and Southern blot analysis identified VIM-1-containing plasmids; estimated sizes were 65 kb (*E. coli* GN531), 50 kb (*E. cloacae* GN738), and 30 kb (*E. cloacae* GN719 and GN825).

In conclusion, VIM-1 was found among *Enterobacteriaceae* from 3 geographically distant nosocomial units in Ontario, Canada. Although *E. cloacae* strains were clonally related, there were no clear epidemiologic links between these patients, suggesting that the clone or resistance gene maybe circulating in the province on a greater scale than believed. Emergence of *E. coli* ST131, a pandemic multidrug-resistant clone that causes predominantly community-onset infections (*7*), and produces simultaneously CTX-M-15 and VIM-1, could be a serious threat for the dissemination of these drug-resistance elements.
